# Modelling Bovine Granuloma Formation In Vitro upon Infection with *Mycobacterium Avium* Subspecies *Paratuberculosis*

**DOI:** 10.3390/vetsci6040080

**Published:** 2019-10-12

**Authors:** J. Hunter Rice, Margaret M. McDaniel, Alyson Holland, Shigetoshi Eda

**Affiliations:** 1 Department of Microbiology, University of Tennessee Knoxville, Knoxville, TN 37996, USA; jrice18@vols.utk.edu; 2 Department of Immunology, University of Texas Southwestern Medical Center, Dallas, TX 75390, USA; Margaret.Mcdaniel@UTSouthwestern.edu; 3 Office of Vital Statistics, Division of Vital Records and Statistics, Tennessee Department of Health, Nashville, TN 37243, USA; alyson.holland@tn.gov; 4 Department of Forestry, Wildlife and Fisheries, University of Tennessee Institute of Agriculture, 1231 Joe Johnson Drive, Knoxville, TN 37996, USA

**Keywords:** granuloma, mycobacterium, paratuberculosis, modelling, pathogenesis, Johne’s

## Abstract

*Mycobacterium avium* subspecies *paratuberculosis* (*Map*) causes chronic granulomatous disease in cattle and ruminant livestock, causing substantial economic losses. Current vaccines delay clinical signs but cannot train the immune system to fully eradicate latent *Map*. During latency, *Map* uses host defenses, cage-like macrophage clusters called granuloma, as incubators for months or years. We used an in vitro model to investigate the early coordination of macrophages into granuloma upon *Map* infection over ten days. We found that at multiplicities of infection (MOI; *Map*:macrophages) of 1:2 and below, the macrophages readily form clusters and evolve pro-inflammatory cytokines in keeping with a cell-mediated immune response. At higher MOIs, viability of host macrophages is negatively impacted. At 1:4 MOI, we quantified viable *Map* in our model and confirmed that intracellular *Map* reproduced over the first five days of infection. Host cells expressed Type 1-specific cytokines, and *Map*-infected macrophages displayed reduced motility compared to *Map*-exposed, uninfected macrophages, suggesting an important role for uninfected macrophages in the early aggregative response. Reported is the first in vitro JD granuloma model capturing *Map* and macrophage viability, size distribution of resulting clusters, motility of monocyte-derived macrophages, and cytokine response during clustering, allowing quantitative analysis of multiple parameters of the *Map*-specific granulomatous response.

## 1. Introduction

Johne’s disease (JD) is a chronic wasting illness of cattle and wild ruminants originally associated with *Mycobacterium avium* subspecies *paratuberculosis* (*Map*) in the early 20th century [1]. *Map* belongs to a genus of recalcitrant bacterial pathogens and shares a common strategy for cellular invasion and proliferation with the human pathogen *M. tuberculosis*. Like other mycobacteria, *Map* possesses a thick waxy cell wall and a slow growth rate, conferring a natural phenotypic resistance to antibiotics and host immune defenses [2,3]. The microbe’s hardy nature further contributes to its epidemiological success by enabling it to persist for up to one year in soil samples [4]. Because of this, and despite the long history of studying mycobacteria and *Map* in particular, global control measures have not successfully curtailed *Map* prevalence in this century since the discovery of its veterinary significance. The prevalence of JD in cattle in Australia, New Zealand, Europe and the U.S. in some studies was estimated to range from 10% to 60% [1,5,6]. In recent decades, it has been possible to calculate the financial toll of JD on the dairy industry in the US, specifically where the USDA reported that 68.1% of dairy herds in the U.S. are contaminated with *Map*, and the most recent report concluded that actual herd-level prevalence of *Map* may be higher than 90% [7]. Here, the annual economic impact of JD on the dairy industry alone has been estimated at 220 million US dollars [8], while damages to the agricultural industry as a whole may be in excess of 1.5 billion [9].

Current control efforts rely on rapid serological tests to identify infected animals before they are euthanized [10,11,12,13]. Unfortunately, there is no cost-effective treatment for JD-affected cattle [14]. Vaccination is a promising alternative for widespread low-cost prophylaxis, but recent studies of whole-cell attenuated vaccines indicate they have questionable efficacy at preventing new infections and intermittent bacterial shedding [15,16]. Furthermore, because of the unique method by which *Map* invades and colonizes its host, attenuated whole-cell and subunit vaccines often fail to illicit the cell-mediated response required to control the early infection, inducing a dominant humoral response instead [17,18]. Not only does this fail to control the pathogen, the humoral response may damage the host and lead to *Map* spread.

Proliferation in macrophages is a commonality shared by most pathogenic mycobacteria and is central to their immunopathology. *Map* is unique in that it invades the terminal portion of the bovine small intestine, where it is taken up into mucosal macrophages [19,20,21]. Unable to efficiently degrade the bacilli, infected macrophages sequester *Map* into closed microenvironments called granuloma, which are persistent clusters of macrophages joined by tight junctions and surrounded by a cortex of uninfected macrophages and activated lymphocytes. Recently, a subset of T-lymphocyte with helper and effector roles called a γδ T cell has been implicated in the development and maintenance of granuloma induced by pathogenic mycobacteria. Here, they are early responders to the site of granuloma development and release interferon (IFN)-γ, a pro-inflammatory cytokine that specifically favors induction of a Type 1 (cell-mediated) immune response [22,23]. Separate studies have demonstrated that animals capable of inducing such a Type 1 response to *Map* without concurrent induction of a humoral (Type 2) response could control, if not clear, the infection [24,25]. These findings suggest an immunological paradigm for successful defense against *Map* invasion, and to better understand the events that lead to clearance, a modelling approach is required.

Selecting a model system for studying immunopathology ultimately requires a tradeoff between the realism of an in vivo system and the efficiency, tractability, and reproducibility of an in vitro system. Mycobacterial pathogens pose additional challenges to in vivo approaches in that (1) granulomatous responses vary widely between model organisms [26], (2) granulomas display great heterogeneity, even within the same animal [27], and (3) time-scales for mycobacterial infection are significantly longer than for those of other bacterial pathogens. For these reasons, in vitro models and macrophage infection assays have gained interest as tools for dissecting the *Map*-specific host-pathogen interaction, inspired largely by work done previously on *M. tuberculosis* [28,29,30]. However, we believe that previous in vitro attempts have failed to capture aspects of the progression of granuloma development that occurs in natural *Map* exposure. In this study, we isolated the chemotactic, aggregative behavior of *Map*-exposed macrophages as a reporter for successful establishment of an infection tightly controlled by a Type 1 cell-mediated immune response. We propose this model not only as a tool for interrogation of early *Map* immunopathology, but also as a potential screening system for exogenous immunomodulators affecting cell-mediated, innate immunity to *Map*.

## 2. Materials and Methods

### 2.1. Blood Sample Collection

Blood samples were obtained by venipuncture from young female calves (less than 2 months old) at the University of Tennessee Little River Animal and Environmental Unit. The farm has no history of JD. The protocol was approved by the University of Tennessee Institutional Animal Care Use Committee (Protocol: #2288-0714).

### 2.2. Preparation and Separation of PBMC Culture

Assays of granuloma formation were conducted using bovine monocyte-derived-macrophages (MDMs). Blood collected using ethylenediaminetetraacetic acid (EDTA) (Fisher Bioreagents, Pittsburgh, PA, USA) as anticoagulant was subjected to discontinuous gradient centrifugation with iodixanol solution OptiPrep™ (1.320 g/mL) from Axis-Shield (Dundee, Scotland) and Hank’s Balanced Salt Solution (HBSS) (GE Healthcare, Chicago, IL, USA) as diluent, according to the manufacturer’s instruction. The density of the blood was adjusted with a working solution of OptiPrep in HBSS (1.205 g/mL final) to 1.095 g/mL and layered under a barrier solution (OptiPrep in HBSS, 1.078 g/mL final). At the top of the column, 2 mL HBSS (1.006 g/mL) was layered to prevent cells sticking to the tube along the meniscus. The column was spun at 700× *g* for 20 min with minimum acceleration, after which the buffy coat was collected from the interface between the barrier and the HBSS layer. Peripheral blood mononuclear cells (PBMCs) were washed three times with HBSS, then suspended in Roswell Park Memorial Institute 1640 medium (MP Biomedicals, Santa Ana, CA, USA) with 10% fetal bovine serum (GE Healthcare, Chicago, IL, USA) (RPMI-FBS) and 2 ng/mL granulocyte-macrophage colony-stimulating factor (GM-CSF) (Corning, Tewksbury, MA, USA). Cells were incubated in an untreated T-75 culture flask (Corning, Tewksbury, MA, USA) overnight at 37 °C, 5% CO_2_ to allow for macrophage differentiation and adherence.

After incubation, the medium was removed along with non-adherent PBMCs, which were pelleted at 300× *g* and suspended in fresh RPMI-FBS. Adherent cells were washed twice with phosphate-buffered saline (PBS; pH 7.6) before being treated with 5 mL of the trypsin-alternative Accutase (Life Technologies, Carlsbad, CA, USA) for 15 min at 37 °C. Once cells were detached, they were diluted in an equal volume of RPMI-FBS supplemented with 1 mM EDTA to delay reattachment. After centrifugation at 300× *g*, the pellet was suspended in 1 mL RPMI-FBS and the adherent cells were quantified using a hemocytometer before being diluted to a concentration of 2 × 10^5^ cells/mL. MDMs in this state were used directly in model setup.

Non-adherent PBMCs were quantified and placed in the same T-75 flask (where a minority of adherent MDMs remained), and a volume of *Map* was added to achieve a multiplicity of infection of 1:1 (*Map*:non-adherent cells). These cells were incubated at 37 °C, 5% CO_2_ for 24 h, then removed and centrifuged at 300× *g*. The supernatant was collected, passed through a 0.22 µm filter, and labeled as conditioned medium of *Map*-exposed non-adherent cells.

### 2.3. Culturing and Preparation of Map Strains

*Map* strain K-10 was cultured at 37 °C in Middlebrook 7H9 broth supplemented with 10% oleic acid-albumin-dextrose complex and 1 g/L Mycobactin J (Allied Monitor, Fayette, MO, USA), with sub-culturing every 2 weeks. *Map* grew as a pellicle along the surface of the culture medium.

For the infection study, a 1 mL aliquot of homogeneous *Map* culture was collected and treated for aggregates by sonication, subjecting the suspension to 30 repetitions of impulses less than 1 s in duration on the lowest power setting. Remaining aggregates were spun down at 2000× *g* and the supernatant was taken. The *Map* concentration was estimated by optical density measurement (in-house comparison of optical density at 600 nm and colony forming unit (CFU) of *Map* K10 shows a linear relationship between optical densities of 0.01 and 1.0) before being diluted to the appropriate concentrations for infection.

For cytokine expression analysis, *Map* enumeration, and macrophage viability and motility analysis, *Map* was added to RPMI-FBS at a final concentration of 5 × 10^4^ cells/mL. For cluster counting experiments and for the calculation of the ratio of infected to uninfected macrophages, *Map* concentrations of 5 × 10^4^, 10^5^, 2 × 10^5^, and 4 × 10^5^ cells/mL were used.

### 2.4. Setup and Maintenance of In Vitro Model

The wells of a Costar^®^ tissue culture treated 24-well plate (Corning, Tewksbury, MA, USA) were inoculated with 1 mL of the adherent cell suspension, and the cells were left to acclimate overnight. After the macrophages were allowed to adhere, the medium from the wells was removed along with contaminating lymphocytes and replaced with 1 mL of fresh RPMI-FBS with concentrations of *Map* bacilli corresponding to an MOI of 1:4, 1:2, 1:1, or 2:1 (*Map*: MDMs).

Every three days, 900 µL of the 1 mL culture medium was removed from the wells and exchanged for conditioned media harvested from non-adherent lymphocytes cultured with *Map* (MOI of 1:1) for 24 h. Medium from wells serving as negative controls was changed with medium conditioned by lymphocytes from the original PBMC populations before GM-CSF exposure and cultured without *Map*.

To visualize the interaction of lymphocytes directly with MDMs in early granuloma-like clusters, preliminary iterations of the model involved reintroduction of non-adherent PBMCs into the granuloma assay on day 0 at a 1:1 ratio with MDMs. Before model setup, MDMs and non-adherent lymphocytes were separately stained with fluorescent membrane dyes 1,1′-dioctadecyl-3,3,3′,3′-tetramethylindocarbocyanine perchlorate (DiI) (Thermo Fisher Scientific, Waltham, MA, USA) and DiI-derivative DiB (Biotium, Freemont, CA, USA), respectively. *Map* cells were stained with cytoplasmic dye carboxyfluorescein succinimidyl ester (CFSE) (BioLegend, San Diego, CA, USA) before infection.

### 2.5. Monitoring Rate of Aggregation

Each day post-infection, 10% of the center of each well (containing approximately 2 × 10^4^ host MDMs on day 0) was imaged using an EVOS FL auto scanning microscope (Thermo Fisher Scientific, Waltham, MA, USA) to create a scanned image at 10× magnification. These images were then analyzed using the software ImageJ [31] by subtracting the background, converting the image to binary, and sorting the clusters of cells by size, circularity, and number. The result was used to quantify the number of cells still adherent, the differences in morphology between infected and uninfected cells, and the number of larger, granuloma-like clusters present.

### 2.6. RNA Isolation and Quantification

Cells were harvested for reverse transcriptase-quantitative polymerase chain reaction (RT-qPCR) from uninfected and infected wells on days 0, 2, 5, and 7. The 1 mL volume was removed from each well and replaced with 150 µL RNAlater (Qiagen, Valencia, CA, USA). The plate containing the harvested wells was then wrapped in parafilm and stored at 4 °C until day 7 post-infection. On day 7, cells in RNAlater were lysed by adding 450 µL RNeasy lysis buffer and pipetting to homogenization. Total RNA was isolated using an RNeasy Plus Kit (Qiagen, Valencia, CA, USA) and quantified via Nanodrop spectroscopy (Thermo Fisher Scientific, Waltham, MA, USA). A normalized mass of RNA was transferred to an RNA-to-cDNA reaction mix (Life Technologies, Carlsbad, CA, USA), and cDNA synthesis carried out in a C1000 Thermocycler (BioRad, Hercules, CA, USA) at 37 °C for 2 h. The resulting ss-cDNA was used for SYBR-green qPCR (Life Technologies, Carlsbad, CA, USA), and the relative change in cytokine expression was determined by Δ-Δ- $C_{t}$ analysis using actin as internal control [32]. Primers used for RT-qPCR are summarized in Table S1.

### 2.7. Measuring Viability of Host Cells

On days 0, 2, 5, and 7, cells were stained using a LIVE/DEAD cell imaging kit (Life Technologies, Eugene, OR, USA). Using the EVOS FL auto microscope, 10% of the center of each well was scanned with green and red fluorescence channels for live and dead stains, respectively. The scanned image was analyzed using the ImageJ software by separating the color channels, converting to binary, and calculating the number of stained particles per channel. The results were used to calculate the viability of adherent macrophages.

### 2.8. Map Enumeration and Viability

On days 0, 2, 5, and 7, internalized *Map* bacilli were harvested from infected wells and subjected to propidium monoazide (PMA)-qPCR to quantify live and total bacteria, as described previously [33,34,35]. To remove extracellular bacilli, the total 1 mL volume of the well was removed, and the adherent cells were washed once with PBS. MDMs were then lysed with 1 mL 0.2% Triton X-100 in PBS followed by a 30-min incubation at room temperature. Next, the lysate was removed to a 1.5 mL tube and centrifuged at 9300× *g* for 5 min, after which the supernatant was replaced with PBS and the suspension homogenized.

Each of the 1 mL samples was separated into two aliquots of 500 µL. One aliquot was subjected to treatment with 12.5 µL PMA, while the other followed the same incubation pattern without the addition of PMA. All samples were incubated in the dark for 5 min, then placed on ice under a 650-watt lamp at 20 cm for two minutes. The incubation pattern was repeated after the addition of another 12.5 µL PMA for a final concentration of 50 µM.

Bacilli were pelleted at 9300× *g* for 5 min, washed, and subjected to genomic DNA purification using a DNeasy kit and protocol (Qiagen, Valencia, CA, USA), with a bead-beating step added for initial lysis and homogenization of *Map* [36]. qPCR on *Map* samples was conducted using a VetAlert Johne’s disease detection kit (Tetracore, Rocksville, MD, USA) with TaqMan probe targeting the *Map*-specific *hspX* gene. To estimate the concentration of *Map* genome copies in samples, a standard curve was constructed from serial dilutions of the supplied *hspX* positive control, and the resulting trendline was used to convert $C_{t}$ values of samples into the number of copies/µL.

### 2.9. Host Cell Motility and Infection Rate

MDMs were infected with CFSE-stained *Map* at a concentration of 5 × 10^4^, 10^5^, 2 × 10^5^, or 4 × 10^5^ cells/mL. On days 0, 2, 5, and 7, six randomly localized 40× images were taken with the EVOS FL auto microscope at each *Map* concentration using green fluorescence and phase-contrast channels (30–50 MDMs per field). Using the superimposed image of these two channels, the total number of macrophages and the number of infected macrophages was calculated to find the ratio of infected to uninfected host cells.

MDMs exposed to CFSE-stained *Map* at MOI of 1:4 were also used to calculate the motility rates of both infected and uninfected MDMs. On day 2 post-infection, a time lapse video was compiled from microscope images taken at 40× magnification from randomly localized areas of the well. Infected cells were distinguished from uninfected cells based on the presence of fluorescent bacteria. The time-lapse image sequences were then analyzed using the ImageJ plugin *wrMTrck*, originally developed to monitor the velocity and displacement of *Caenorhabditis elegans* [37].

## 3. Results

### 3.1. Host Cell Cluster Formation in the Presence of Lymphocyte-Specific Signaling Factors

**Figure 1:**
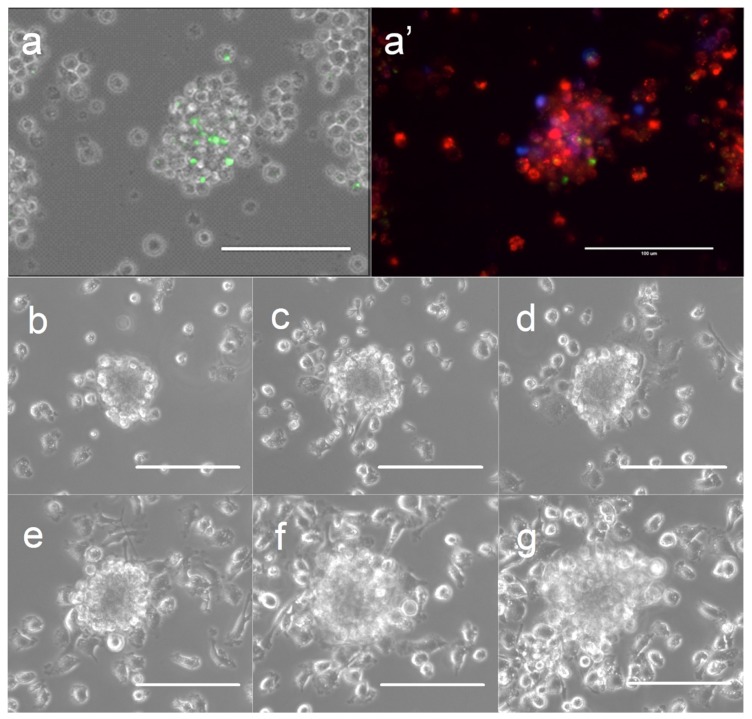
Formation of granuloma-like cell clusters (GLCCs) in vitro. (**a**) GLCC of macrophages and lymphocytes exposed to *Map*. (**a′**) Same GLCC with superimposed fluorescence channels showing macrophages (red), lymphocytes (blue), and internalized *Map* (green). (**b–g**) Phase-contrast images of a single GLCC across the time-course at 1-day intervals (3–8 days post-infection; lymphocyte-free model). The scale is 100 µm.

Clusters of bovine MDMs (termed granuloma-like cell clusters [GLCC] in this paper) began to appear by day 2 post-infection in *Map*-exposed wells while background GLCC in *Map*-free wells remained low throughout the experimental time-course. To confirm that the clusters observed were persistent cellular superstructures rather than transient spatial associations of cells, several individual GLCCs were tagged and tracked throughout the time course (representative shown in Figure 1b–g). In MDM cultures supplemented with non-adherent PBMCs, GLCCs were composed of infected and uninfected MDMs and a small number of lymphocytes surrounding the macrophage cluster (representative shown in Figure 1a,a′).

**Figure 2:**
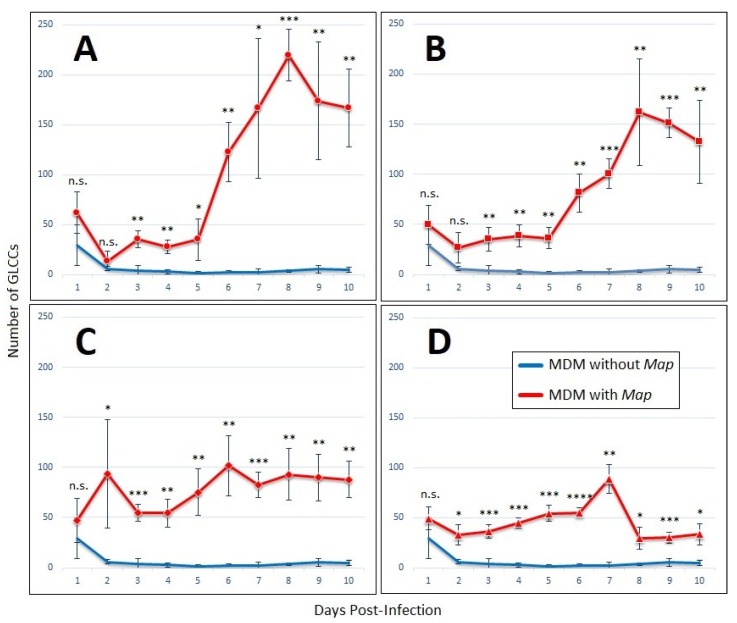
GLCCs form in the presence of lymphocyte-specific signaling factors alone. Graphs of GLCC formation over ten-day experiment with MDMs cultured without non-adherent PBMCs, with conditioned medium from *Map*-exposed lymphocytes, after exposure to *Map* at an MOI of (**A**) 1:4, (**B**) 1:2, (**C**) 1:1, or (**D**) 2:1. The bars are standard deviation. * *p* < 0.05; ** *p* < 0.01; *** *p* < 0.001; **** *p* < 0.0001; n.s. = not significant by student’s t-test (n = 4).

The number of GLCCs formed over ten days was enumerated by analyzing images capturing 10% of the total MDM population (Figure 2). In wells infected at *Map* MOIs of 1:4 and 1:2, there was a lag in cluster formation from day 2 until day 5, after which persistent clusters began forming rapidly until they peaked at day 8 post-infection. Both the rate of formation and total count of GLCCs was higher at MOI of 1:4 than at 1:2 (Figure 2A,B)

**Figure 3:**
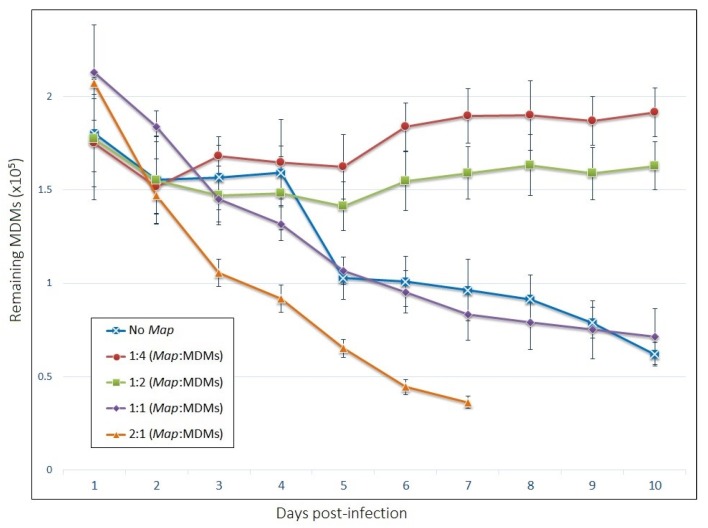
The viability of host MDMs in our model depends on the multiplicity of *Map* exposure. The number of adherent cells remaining by day post-infection. The bars are standard deviation (n = 4).

At MOI of 1:1, the GLCC count remained relatively steady throughout the experiment, however, GLCCs from these wells possess a different appearance compared to those infected at lower MOI. Individual and GLCC-incorporated MDMs appear necrotic (darkened cytoplasm with indistinct/irregular cell membranes). At the highest MOI of 2:1, the necrotic appearance of cells is more apparent, and the viability of cells declines rapidly to the point the accumulation of cellular debris prevented quantification of living cells past day 7 (Figure 3). Thus, an MOI of 1:4 was selected for cytokine profiling and motility experiments due to the robust clustering response that occurred at this *Map* concentration as well as the increased stability of GLCCs and prolonged viability of MDMs.

### 3.2. Viability of Host Mdms and Infection Rate

The numbers of MDMs remaining adherent across the time-course are shown in Figure 3. Live/Dead staining of MDMs indicate that about 95% of adherent cells were viable at each time point (Figure S1). Interestingly, cells infected at the two lower MOIs displayed increased longevity compared to uninfected cells, which saw a population reduction of 50% within seven days post-infection. Cells infected with an MOI of 1:1 experienced a death rate over the ten-day period comparable to uninfected cells, while those infected at 2:1 died significantly more quickly.

**Figure 4:**
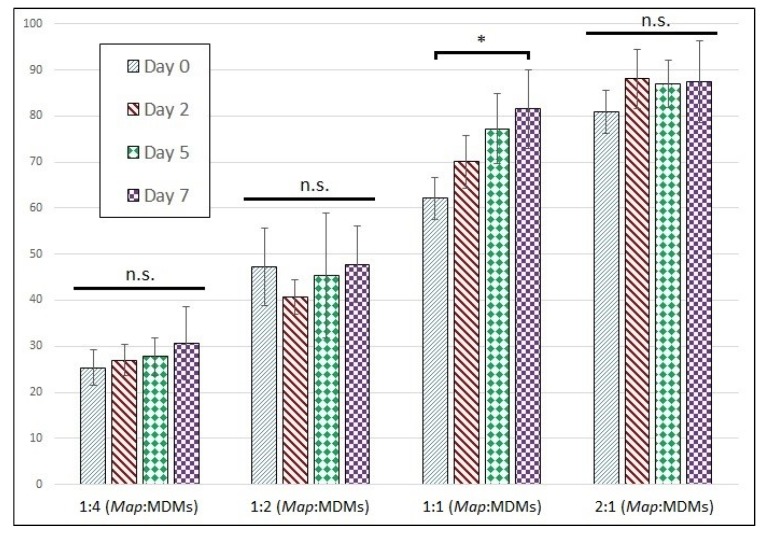
Proportion of infected MDMs remains stable across time-course. Proportion of infected MDMs with intracellular CFSE-stained *Map* expressed as a percentage of total cells. The bars are standard deviation. * *p* < 0.05; n.s. = not significant by student’s t-test (n = 6).

Moreover, interestingly, the prolonged viability of cells in *Map*-exposed wells appears to be a property of both uninfected and infected populations from within the same well. The proportion of host cells infected with *Map* by day is shown in Figure 4. These data indicate the proportion of infected cells does not change significantly across the time-course for any of the wells except at a *Map* MOI of 1:1, which experienced a small but significant increase in infection rate by day 7. Based on the result from MDMs infected at an MOI of 1:4, both the infected and uninfected populations from within the same well must have similar death rates, even as the death rate varies significantly between different MOIs.

### 3.3. Host-Cell Behavior and Growth of Intracellular 

#### 3.3.1. Cytokine Expression Profile Changes in Infected Mdms

**Figure 5:**
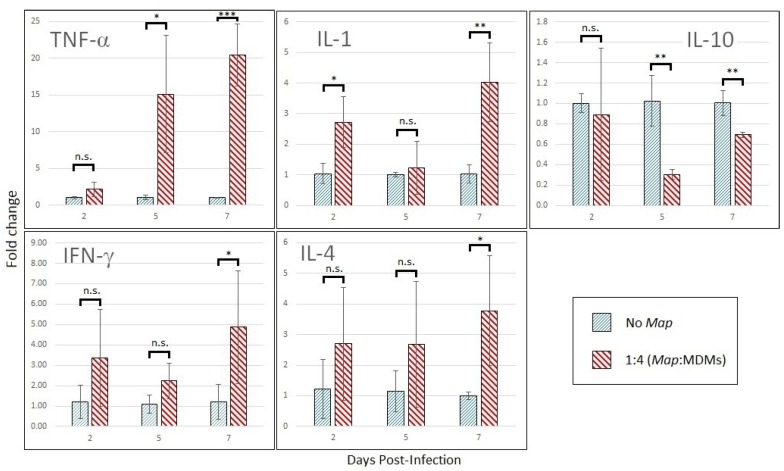
Cytokine profile shift of *Map*-infect MDMs across the time-course. Cytokine expression of MDMs infected with *Map* at an MOI of 1:4 compared to same-day uninfected MDMs. RT-qPCR was run using actin as internal control, and the data was converted to fold-change by Δ-Δ- $C_{t}$ method. The bars are standard deviation. * *p* < 0.05; ** *p* < 0.01; *** *p* < 0.001; n.s. = not significant by student’s t-test (n = 3).

MDMs infected at a *Map* multiplicity of 1:4 display transcription-level shifts in cytokine expression marked by upregulation of TNF-α by 15-fold by day 5 post-infection with concurrent downregulation of IL-10 (Figure 5). Intermittent upregulation of IL-1 was observed over the time course. The expression analysis also picked up late induction of T-cell-produced IL-4 and IFN-γ by day 7 post-infection. MDMs infected at 1:1 MOI show similar trends in cytokine expression but increase the production of IL-10 (Figure S2; data in duplicate).

#### 3.3.2. Host-Cell Motility

**Figure 6:**
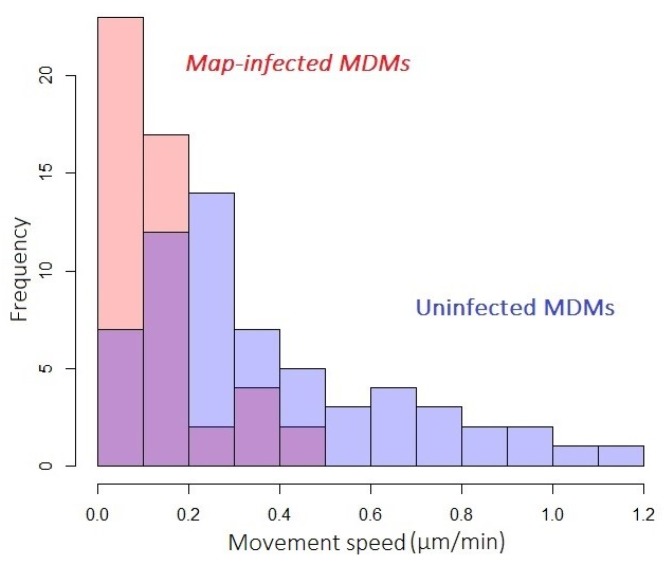
*Map*-infected MDMs show decreased movement speed. Histograms describing the motility of populations of *Map*-infected (red) and uninfected (blue) MDMs exposed to *Map* at MOI of 1:4. Overlap of histograms in purple.

The distribution of movement speeds displayed by populations of infected or uninfected cells exposed to *Map* at an MOI of 1:4 is shown in Figure 6. Observations were made on day 2 post-infection, whereupon infected MDMs displayed significantly lower motility compared to uninfected. Motility data from infected and uninfected populations fell into non-parametric, positively skewed distributions (Shapiro–Wilk *p*-values of 1.7 × 10^−5^ and 1.1 × 10^−4^, respectively) that were statistically distinct by the Kolmogorov–Smirnov test (*p*-value of 7.1 × 10^−8^).

#### 3.3.3. *Map* Viability

**Figure 7:**
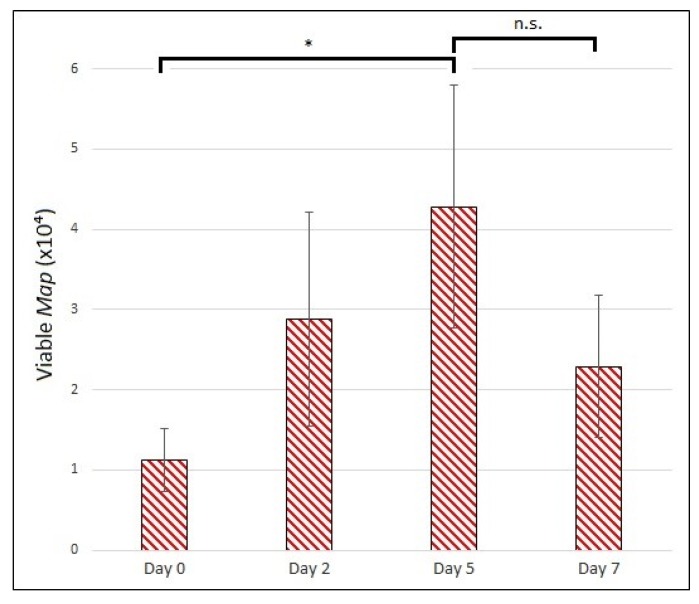
Enumeration of viable, internalized Map from in vitro granuloma model. Viable Map bacilli harvested and quantified on days 0, 2, 5, and 7 p.i. MOI of 1:4. The bars are standard deviation. * *p* < 0.05; n.s. = not significant by student’s t-test (n = 3).

Using the established technique for PMA-modified qPCR of *Map*, we were able to detect viable bacilli growing intracellularly within the MDMs in our model (Figure 7). We observed a statistically significant increase in viable *Map* from day 0 to day 5 post-infection (*p*-value of 0.025), indicating the intracellular population of *Map* can reproduce under the conditions of our model. The mean number of viable *Map* dropped between day 5 and day 7, but this difference was not significant (*p*-value of 0.12).

## 4. Discussion

One of the major hurdles to understanding *Map* (and by extension, mycobacterial) infection lies in discriminating immune effects that benefit the host from those that benefit the pathogen. The problem is greatest at the onset of latency, during which the generation of a granuloma may be interpreted as either host-interest-oriented, intended to prevent the spread of infection by containing a foreign agent, or pathogen-interest-oriented, intended to provide the ideal host environment for bacterial proliferation. Assays of inflammatory cytokine production provide insufficient data to distinguish the two over time periods relevant to *Map* pathology and may even underestimate the timescale required by *Map* to subvert innate immune functions that might otherwise impede *Map* latency. Misinterpretation of cytokine responses meant to aid in *Map* resuscitation as instead being host-protective, or vice-versa, may confound efforts to understand both the order of events necessary to establish latent infection as well as the optimal target for disruption of this process. Our model employs a secondary output that correlates the protective or detrimental effects of cytokine signaling events with infection progression temporally. Such a model, while lacking the degree of realism achieved in vivo, offers far more information than a cytokine assay alone. To this end, our simple in vitro model of GLCC formation provides tractable tools for interrogating the early host-pathogen interaction during *Map* infection.

Basic *Map*-infection studies of bovine MDMs have helped elucidate the variety of natural immunological responses to the bacterium [38]. Pro-inflammatory cytokines interleukin (IL)-1 [39,40,41] and tumor necrosis factor (TNF)-α [42,43,44], anti-inflammatory cytokines IL-10 [40,43,44,45] and IL-6 [40,41,45,46], and chemokine macrophage inflammatory protein (MIP)-1 [45] have all been demonstrated to be differentially regulated upon *Map*-exposure. However, these assays have been performed at MOIs ranging from 2:1 up to 10:1 *Map* bacilli per host cell, and it is questionable whether that high an environmental load of mycobacteria represents the start of a natural infection. Furthermore, a study of *Map* infectious dose observed increased rates of apoptosis and necrosis among macrophages exposed at 10:1 MOI and above, while exposure at 1:1 saw no significant increase [47], indicating that somewhere between multiplicities of 1 and 10 lies an inflection point at which the *Map* dose overwhelms the response of the primary host cell. MDM-infection assays using 2:1 or higher MOIs report upregulation of IL-10, IL-6, and tumor growth factor (TGF)-β, cytokines associated with suppression of cell mediated immunity [40,42,43,44,45,46]. Accordingly, IL-12, TNF-α, and TNF receptor, important signaling molecules in transduction of a Type 1 response, are routinely downregulated. These results contradict the observations made of this model, where TNF-α and IL-1 were upregulated, but IL-10 was downregulated. Thus, the in vitro model described here captures features of a host-interest-oriented Type 1 response that would lead to granuloma formation and maintenance in vivo.

It is worth noting the late upregulation of IL-4 and IFN-γ in our model, however, as we witnessed their induction in populations subjected to selection for MDMs, and both IL-4 and IFN-γ are primarily produced by T lymphocytes [48,49,50]. Our model likely contains a small proportion of contaminating lymphocytes, also indicated by the fact that RNA for these T-cell-specific cytokines was present at average concentrations of 2.7 (IFN-γ) and 4.6 (IL-4) orders of a lower magnitude on day 0 than that of the next lowest-transcribed cytokine, TNF-α (average C_t_ = 24.8). Expression of these cytokines usually accompanies activation of naïve T cells by dendritic cells (DCs) in the lymph nodes [51]. However, recent evidence indicates that activation of naïve T cells may occur ectopically at the site of inflammation in mucosal tissues [52,53,54] and that non-DC antigen-presenting macrophages play a greater role in T cell activation than previously thought [55,56]. Given the late timing of IL-4 and IFN-γ regulation, it is possible that this shift occurred as a result of T cell activation within the in vitro system.

In contrast, the period allowed for *Map* exposure of non-adherent PBMCs to generate conditioned medium was too short (24 h) to expect robust T cell activation through the classical route. The shorter incubation specifically targets activation of innate lymphoid populations like γδ T cells, which represent a significantly larger proportion of circulating PBMCs in calves than in humans of any age (34% measured at 2 months old [57]). These T cell variants possess a restricted T cell receptor repertoire with activity toward mycobacterial phosphoantigens [58] and may begin effector functions without the need for MHC-dependent antigen presentation or clonal expansion [59], reducing their response time from days to hours. In JD, *Map*-antigen responsiveness of γδ T cells is more robust in subclinical compared to active infection, suggesting a role in *Map* constraint [60]. Like CD4+ T cells, γδ variants may develop to produce either Type 1 (IFN-γ) or Type 2 (IL-4, IL-10, TGF-β) cytokines in response to different stimuli [61], adding to the complexity of their interaction with *Map*.

IFN-γ and IL-4 are major signaling intermediates in the development of opposing immune responses and lead to divergent outcomes in *Map* pathology. IFN-γ expressed by helper and γδ T cells stimulates cell-mediated inflammation and induces macrophages to undergo autocrine and paracrine TNF-α signaling required for granuloma development [62]. Conversely, IL-4 produced by innate and adaptive T cells stimulates a Type 2 response and has been implicated as a costimulatory cytokine along with IL-10 to suppress Type 1 immunity during *Map* infection [63,64]. These T cell responses have parallels in macrophage subtypes, with Type 1 cytokines driving classical (M1) activation of macrophages while Type 2 cytokines favor alternative (M2) activation. M1 polarization primes macrophages for a variety of pro-inflammatory cell-mediated responses, among them granuloma formation. M2 polarization, meanwhile, accompanies humoral immunity, is favored by IL-10 stimulation, and redirects uninfected MDMs from granuloma formation to wound repair [65]. GLCCs formed under conditions favoring M2 polarization more closely model activation of latent *Map* than they do the early stages of latency, while the latter is what we aim to simulate. Moreover, an investigation of granulomatous intestinal lesions of JD-positive cattle found that macrophages associated with focal paucibacillary granuloma were predominantly of M1 subtype, while diffuse, multibacillary granuloma had higher proportions of M2 macrophages, suggesting that M1 macrophages are more effective in spatial control of granuloma and containment of *Map* [66]. It remains to be seen if this paradigm extends to *Map* eradication, or if a downstream M1–>M2 switch may result in re-emergence even from well maintained, M1-predominating granuloma.

The timing and magnitude of signaling events underlying macrophage differentiation are likely controlling factors in granuloma development and curation, so it is also worth noting that TNF-α and IL-10, key regulators in this balance, did not experience their differential regulation until day 5 post-infection, when viable *Map* number peaked. This is significantly longer than the duration of previous *Map*-infection assays, most of which tested host cell expression at or before 24 h post-infection [39,40,44,45,46,67]. Based on these findings, it seems the immediate immunological response to *Map* provides insufficient data to relate cytokine expression with granuloma outcomes. For mycobacterial pathogens, in vitro models of granuloma formation offer a promising alternative to macrophage-infection studies because they convert a complex process into a simple output (cluster formation) for monitoring the progress of the infection. In this way, researchers hope to characterize the immunopathological changes consistent with productive infection leading to latency. *In vitro* granuloma models have been developed to study *M. tuberculosis* [29,68,69,70,71,72,73,74,75,76], *M. leprae* [77], *M. bovis* [78], *M. massiliense* [79], and *Map* [44]. Unique iterations of the *M. tuberculosis* model were used to investigate resuscitation from latency [69,76], consequences of macrophage polarization [71], and effects of cytokine suppression on granuloma development [74]. Granuloma models of other mycobacterial pathogens provide useful comparisons to *M. tuberculosis* with respect to bacterial survival, host response, and optimum MOI for cluster formation. Predictably, the MOI required to induce expression of Type 2-specific cytokines associated with reemergence is smaller in vitro with *M. tuberculosis* than other mycobacteria, as is the MOI associated with granuloma induction (1:200 [76]). Modelling *M. bovis*, a zoonotic cause of tuberculosis, researchers used MOIs of 1:1 to 5:1 to induce granuloma formation, seeming to contradict the observation that more virulent mycobacteria require lower MOI in these model systems [78]. However, the authors noted that they were unable to see clusters form on tissue culture plates using ultra-low attachment surfaces for cluster counting. If the rate of cluster formation in their model is as dependent on recruitment of uninfected MDMs as it appears to be in our model, the high multiplicity of *M. bovis* may have delayed cluster formation on tissue culture plates by depleting these highly motile, exposed-but-uninfected MDMs, as we observed at higher *Map* multiplicities.

Dependence of both aggregation rate and Type 2 signaling on MOI illustrates the influence of bacterial burden on the M1/M2 balance in these model systems. Furthermore, this suggests that the conditions of the in vitro model may be manipulated to reproduce aspects of either latent or active *Map* infection. For this reason, we chose to replace non-adherent lymphocytes with their soluble signaling factors, as we observed lymphocyte populations to be less robust in vitro regardless of *Map* exposure and did not want their higher death rate to affect the M1/M2 balance of MDMs. A previous model of granuloma induction by *Map*-infection of PBMCs [44] observed aggregative behavior between 1:33 and 1:8 MOI (*Map*: total PBMCs; about 1:6 and 1:1.5 *Map*: MDMs, respectively, if monocytes account for ~18% of PBMCs [80]). However, expression analysis of macrophages was performed at an MOI of 10:1 (*Map*: MDMs), whereupon macrophages increase the production of cytokines TGF-β1 and IL-10. Expression of TNF-α was not seen to rise over the time course. Overall, this indicates an M2-expression phenotype similar to that observed upon *M. tuberculosis* exposure but does not correlate the M2 profile with in vitro aggregation. Our model identifies an upper limit to MOI under which MDMs form persistent clusters while evolving pro-inflammatory cytokines. This threshold represents the tipping point between diverging immunopathogenic routes, the point at which deficiencies in *Map* virulence will have the greatest effect on model outcomes. Therefore, model designs targeting MOI directly above or below this point have the most promise discriminating subtle differences between virulent *Map* and attenuated strains possessing vaccine or research potential.

In the management of JD, the need for a low-cost high-efficacy prophylactic option like a prophylactic vaccine cannot be overstated. However, and keeping with its similarity to tuberculosis, vaccines to *Map* do not provide complete and lasting protection. This may be in part due to the unique strategy of mycobacterial pathogens; sequences encoding T-cell specific epitopes of *M. tuberculosis* are among the most conserved within its genome, suggesting a strong induction of adaptive immunity plays an integral role in its pathogenesis [81]. Because mycobacterial vaccines aim to induce immune memory by the same route, they are unlikely to result in complete eradication of latent bacilli. However, innate immunity appears particularly effective at clearing *M. tuberculosis* infection in its early stage, before an adaptive response may be engaged at all [82], and it is reasonable to believe a similar route for immune cell activation, circumnavigating the pathogens adaptation to adaptive immunity, may lead to clearance of a greater proportion of infecting *Map* as well. Therefore, augmentation of intracellular processes influencing innate immunity poses a promising supplement or alternative to traditional vaccination when it comes to clearance of latent mycobacteria. Our model is uniquely suited for testing exogenous immunomodulators for influence on either the bactericidal or bacteriostatic activity of macrophages.

**Figure 8:**
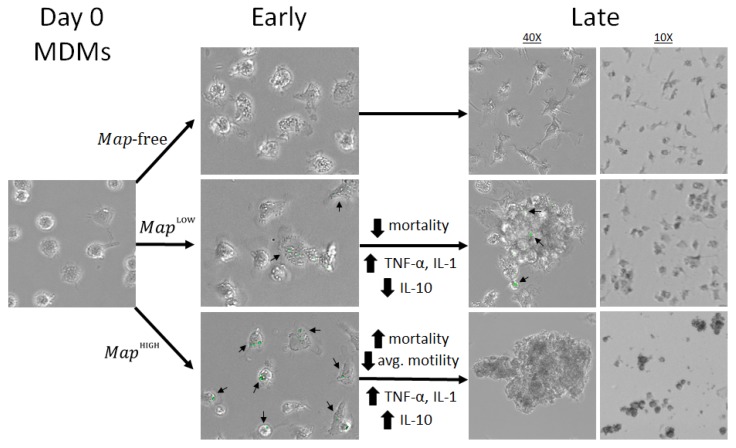
Schematic summary of the findings of this model. *Map* bacilli stained with CFSE (green) and marked with arrows.

Finally, the dynamics of granuloma formation have not been explored in *Map* infection with the resolution reported here. Mathematical modelling presents an emerging opportunity to investigate influences of known intermediates in the granulomatous response on rates of aggregation and viability of internalized *Map*. However, realism in virtual model systems suffers when model parameters must be estimated from multiple unrelated sources. *In vitro* models offer a wealth of information for the characterization of a diverse set of quantifiable parameters of host and pathogen behavior, making them ideal sources for parameterization of math models of infection. A summary of the findings in this study is depicted in Figure 8. For our future work, we intend to develop an *in silico* model of granuloma induction to evaluate and rank the contribution of parameters taken from the in vitro model on *in silico* outcomes.

## Supplementary Materials

The following are available online at https://www.mdpi.com/2306-7381/6/4/80/s1, Table S1: Primer sequences for RT-qPCR, Figure S1: Viability of adherent MDMs by day by LIVE/DEAD fluorescent assay, Figure S2. Cytokine profile shift of MDMs infected with 1:1 MOI of *Map*.
