# Supplementary material for: Modelling Bovine Granuloma Formation In Vitro upon Infection with Mycobacterium Avium Subspecies Paratuberculosis
Source: Vet Sci. 2019 Oct 12;6(4):80. doi: 10.3390/vetsci6040080 (PMC6958389; doi:10.3390/vetsci6040080)
Supplement: Supplementary file 1 [file vetsci-06-00080-s001.pdf]

Supplement

Table S1. Primer sequences for RT-qPCR

Primer Target	Forward Sequence	Reverse Sequence
Actin	ctaaggagaaggggccagtc	tgatggctgtccattcaaaa
Interleukin 1β	ggaaatggcaacctactcca	ttcagtcgtgtccgactctg
Interleukin 4	cagtgctggtctgcttactg	aggatgttcagcgttttgat
Interleukin 10	ctccaagagaggggtgtcta	tgcttcacttttgcatcttc
Interleukin 12	actactcccaaaacctgctg	atcgatctcctcagaagtgc
Interleukin 18	aaagggacctcaaacctttc	ccacaaagctgatgcaataa
Interferon γ	ttcaactactccggcctaac	ggccataagaaccagaaaaa
Tumor growth factor β	ccatactggccctttacaac	gacctccttggcgtagtagt
Tumor necrosis factor α	actcacaggtcctcttcagg	gttgaccttggtctggtagg

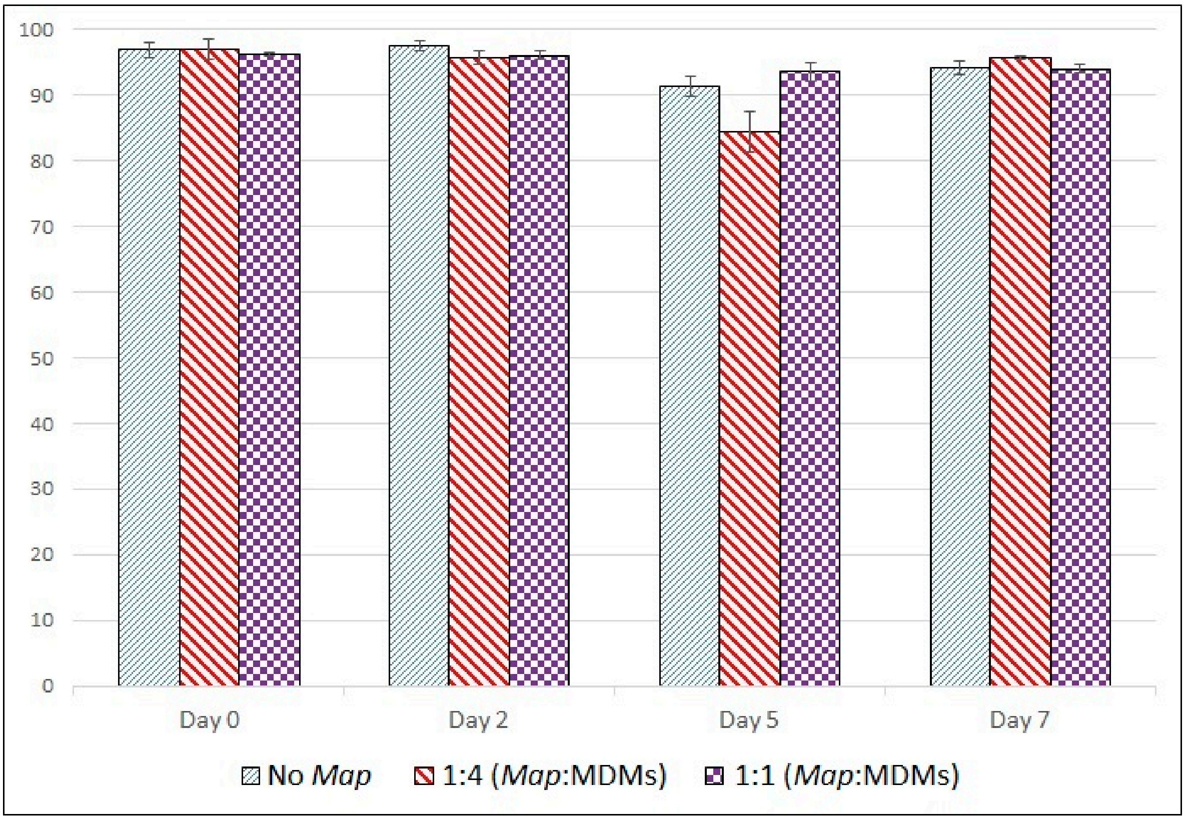

Figure S1. Viability of adherent MDMs by day by LIVE/DEAD fluorescent assay. Expressed as a percentage of total cells. Bars are standard deviation (n = 3).

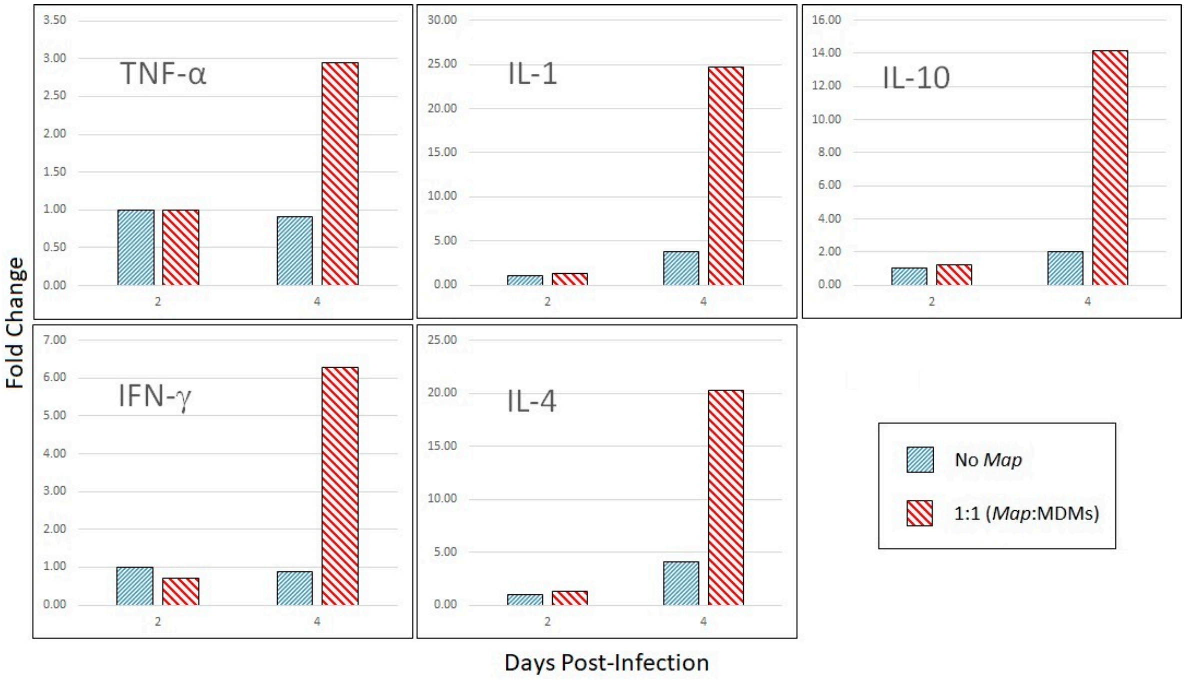

Figure S2. Cytokine profile shift of MDMs infected with 1:1 MOI of *Map*. Cytokine expression of MDMs infected with *Map* at MOI of 1:1 compared to same-day uninfected MDMs. RT-qPCR was run using actin as internal control, and the data was converted to fold-change by Δ-Δ- C_t method. Data collected in duplicate. Results are representative of two experiments.
